# 500 A Hospital-Based School Program May Ease School Reintegration for Pediatric Burn Patients

**DOI:** 10.1093/jbcr/iraf019.129

**Published:** 2025-04-01

**Authors:** Ella Stewart, Lia Thibodaux, Samantha Bonge, Michelle Curtin, Debra Reisinger

**Affiliations:** Wake Forest University School of Medicine; Wake Forest University School of Medicine; Wake Forest University School of Medicine; Wake Forest University School of Medicine; Cincinnati Children’s Hospital and University of Cincinnati College of Medicine

## Abstract

**Introduction:**

Pediatric patients with burns may have difficulty returning to school due to long hospital stays, changes in behavior, weaknesses in cognitive skills, loss of social interaction and support, and increased rates of bullying victimization. Hospital Based School Programs (HBSPs) may promote school reintegration, a key part of psychosocial recovery, by communicating with caregivers, the medical team, and the school of record to plan effective transitions back to school. The specific school supports by burn patients from HBSPs have not been previously studied. We sought to understand how pediatric burn patients who utilize HBSP resources access formal supports of Individualized Education Programs (IEPs) and Section 504 Accommodation Plans (504 Plans) compared to the national average. We also describe the use of Medical Homebound and Medical Report Forms in our sample.

**Methods:**

This study included 41 pediatric burn patients served by a single HBSP across 2021-2022 and 2022-2023 school years. Data included patient demographics, school information, medical diagnosis, and recommended school supports. Analyses examined descriptive statistics with the use of Microsoft Excel and SPSS.

**Results:**

Forty patients received inpatient HBSP supports and 1 was seen in outpatient Burn Clinic. The average inpatient stay was 9.2 days (SD=12.2). Approximately half of the patients were male (n=22). The mean age was 10.1 years (SD=4.6). The majority of patients identified as White (n=26) with the remainder identifying as Black (n=12), Asian (n=2), and Other (n=1). Most patients were Non-Hispanic/Latinx (n=33). Seven patients had pre-existing IEPs (n=5, 12%) or 504s (n=2, 5%). HBSP recommendations included 1 (2%) IEP, 5 (12%) 504 Plans, and 3 (7%) Medical Homebound placements. The HBSP provided 8 (20%) Medical Report Forms and attended multiple school meetings (n=3, 7%) and post-discharge consultations (n=14, 34%).

**Conclusions:**

HBSP supports were well utilized by this cohort of pediatric burn patients, with 504 Plan recommendations made for 12% of our sample, which is higher than the national of 3%. Notably, the pre-HBSP encounter rate was 2%, revealing a higher need for school supports. One percent of patients received an IEP recommendation, and 12% had a pre-existing IEP, which is lower than the national of 14%. Unknown details pertaining to these IEPs could inform future research into specific needs. School reintegration is known to improve with hospital-school communication, which this HBSP provided through recommendations for changes in the school supports (e.g., IEP, 504 Plan), placement (Medical Homebound) and by sharing medical needs via Medical Report Forms or direct meetings.

**Applicability of Research to Practice:**

The goal would be a standardization of school reintegration recommendations for pediatric patients with burns using this knowledge of school supports and future research in this field.

**Funding for the Study:**

N/A

